# One Single Amino Acid for Estimation the Content of Total Free Amino Acids in *Qingkailing Injection* Using High-Performance Liquid Chromatography-Diode Array Detection

**DOI:** 10.1155/2014/951075

**Published:** 2014-03-13

**Authors:** Li Zhang, Xue Wang, Jiankun Su, Haiyu Liu, Zhixin Zhang, Lingling Qin, Cheng He, Long Peng, Mingxing Guo, Xiaoyan Gao

**Affiliations:** Science Experiment Center for Traditional Chinese Medicine, Beijing University of Chinese Medicine, No. 11 North Third Ring Road, Chaoyang District, Beijing 100029, China

## Abstract

*Qingkailing injection (QKLI)*, a modern traditional Chinese medicine preparation, has been widely used in clinics due to its fast and significant efficacy in treatment of high fever. The free amino acids (AAs) were considered to be the most abundant active ingredients indisputably. So developing an accurate and simple determination method to measure the contents of total free AAs in *QKLI* is very crucial. In current study, the accurate and simple method of using one single standard AA for simultaneous quantification of multiple AAs (One for M) in *QKLI* was developed. Particularly, the calculation methods and the robustness of relative correction factors (RCFs) were investigated systematically. No statistically significant difference between these two quantification methods of One for M and classic regression equation was found by the *t*-test (*P*  =  95%, *P* > 0.05). The results showed that the precision (RSD < 4.88%), the robustness (RSD < 4.04%), and the average recoveries (94.11%–107.94%) of this newly proposed method all met the requirements for content determination. This One for M method will provide a scientific reference for the quantitative determination of AAs in other traditional Chinese medicines and their preparations owing to its accuracy and simplicity.

## 1. Introduction

*Qingkailing injection (QKL)*, which is modified from a well-known classic formulation An-Gong-Niu-Huang pill, has become one of the essential Chinese patent medicines for its wide clinical application in the treatment of liver damage, inflammation, viral infections, and cardiovascular diseases, especially hyperpyrexia [1–5]. The formula of* QKLI* is composed by eight medicinal materials or their extracts, in which amino acids (AAs) are the main bioactive components which come from three drugs, including* Margaritifera Concha, Bubal Cornu, *and* Isatidis Radix *[6–9]. By now, the content of total nitrogen derived from free AAs is the key control index in the complex production process of* QKLI*. In the monograph of Chinese Pharmacopoeia (2010 edition), the Kjeldahl nitrogen determination method, a classic method for analyzing the content of total nitrogen, was used to evaluate the total content of AAs in* QKLI* indirectly [10]. Some disadvantages of this nitrogen determination method, including time-consuming, complicated operation and the consumption of large amounts of solvents, are not facilitative to accomplish the determination of the total nitrogen. What is more, this Kjeldahl's method could not distinguish the nitrogen between the free AAs and the binding AAs or the inorganic nitrogen and the organic nitrogen. To ensure the security and the efficiency of* QKLI*, the accurate determination of multiple AAs and evaluation of the content ratio of them are essential. So, developing an accurate and simple determination method of multiple AAs used in production process of* QKLI* and quality control to its preparation is imperative.

Though the determination methods of AAs were reported in many literatures, most of them mainly focused on the optimization of the instruments [11–16], derivatization reagents [17–20], and columns [21, 22]. The method of using one single standard substance for simultaneous determination of multiple AAs (One for M) has not been reported. As we all know, AAs occurring in herbal medicines are mostly in the form of homologues and have similar chemical structures, implying that there are no obvious differences in their derivatization efficiencies and chromatographic behaviors. Therefore, it is more suitable to employ One for M method to simultaneously determine the content of multiple AAs.

Up to now, a number of studies have been reported on the determination of multiple active components in the herbal medicines by One for M [23–30]. But the key node of One for M, namely, the calculation method of RCFs, has not been investigated adequately in previous researches. In our previous study, One for M method was adopted to carry out an assay of seven anthraquinone ingredients in rhubarb [31]. With further research, we found that the effect of intercept and slope of calibration curve on RCFs could not be ignored. So, in this study, we went into depth study on the calculating methods of RCFs and made a comparison among them. Then we selected the best one by investigating the relative deviation of the contents between the methods of One for M and classic calibration curve in ten batches of* QKLI*. Afterwards, the *t*-test was performed to validate the feasibility of the present method. At the same time, the robustness of RCFs was also investigated systemically for enhancing the applicability of the proposed method, including the effects of different instruments, columns, and HPLC conditions.

In our previous work, a HPLC method was developed and validated for the simultaneous determination of twenty AAs in* QKLI* based on a pre-column derivatization with phenylisothiocyanate (PITC) [32]. In current study, fourteen AAs with higher contents and good resolution were chosen for the methodology study, which accounted for about 97% of total free AAs in* QKLI*. Above all, we presented an accurate and fast One for M method for the simultaneous quantification of fourteen AAs with Gly as the internal standard, aiming at realizing a better quality control to* QKLI*.

## 2. Experimental

### 2.1. Instrumentation

Analyses were performed on an Agilent 1100 liquid chromatography system (Agilent Technologies, Palo Alto, CA, USA) comprising a binary solvent delivery system, an online degasser, an autosampler, a column temperature controller and a diode-array detector (DAD) coupled with an analytical workstation (HP, USA), and an Alliance 2695 LC system (Waters Corp, Milford, MA, USA) comprising a quaternary solvent delivery system, a vacuum degasser, an autosampler, a column temperature controller, and a photodiode array detector (PDA) coupled with an analytical workstation (Empower 3). Chromatographic separation was operated on three different chromatographic columns: Diamond C_18_ (4.6 mm × 250 mm, 5 *μ*m), Phenomenex Luna C_18_ (4.6 mm × 250 mm, 5 *μ*m), and Hypersil BDS C_18_ (4.6 mm × 250 mm, 5 *μ*m).

### 2.2. Reagents and Materials

The 14 AAs standards including glycine (Gly), aspartic acid (Asp), alanine (Ala), ornithine (Orn), glutamic acid (Glu), lysine (Lys), arginine (Arg), proline (Pro), valine (Val), tyrosine (Tyr), isoleucine (Ile), leucine (Leu), and tryptophan (Try) (with the purity higher than 99.5%) were all purchased from Xinjingke biotechnology company (Beijing, China). Sixteen batches of* QKLI* were obtained from YaBao pharmaceutical Group Co., Ltd. (Beijing, China).

HPLC-grade acetonitrile was supplied by Fisher Scientific (Fair Lawn, NJ, USA). Phenylisothiocyanate (PITC, lot code: 10113227) was purchased from Alfa Aesar Chemical Co. Ltd (Tianjin, China), and the purity was higher than 97%. Triethanolamine (TEA, analytical grade) was purchased from Fuchen Chemical (Tianjin, China). Other reagents were all analytical grades and obtained from Chemical Works (Beijing, China). Ultrapure water (18.2 MΩ) was generated from Milli-Q water purification (Millipore, France).

### 2.3. Preparation of Standard Solution

Each of the 14 AAs was accurately weighed (Asp 49.94 mg, Glu 99.30 mg, Ser 49.87 mg, Gly 100.82 mg, Arg 50.25 mg, Ala 99.33 mg, Pro 200.29 mg, Tyr 34.67 mg, Val 50.08 mg, Ile 30.35 mg, Leu 99.63 mg, Phe 49.50 mg, Orn 50.48 mg, and Lys 30.23 mg). The stock solution was prepared by dissolving the above 14 standards in 50 mL volumetric flask with 0.1 mol/L hydrochloric acid and stored in refrigerator at 4°C until being used.

### 2.4. Sample Preparation

*QKLI* 2 mL was accurately placed into 25 mL volumetric flask, and then 6 mL of phenylisothiocyanate (PITC) acetonitrile solution (0.1 mol/L) and 6 mL of triethylamine (TEA) acetonitrile solution (1 mol/L) were added. After 60 min of ultrasonic bath (60 Hz), the solution was taken out, cooled to room temperature, and diluted with 50% acetonitrile aqueous solution to 25 mL. 10 mL of the above solution was placed in the separating funnel; after the addition of 10 mL of n-hexane, the mixture was then shaken thoroughly and the underlayer solution was filtered through a 0.45 *μ*m membrane filter for injection.

### 2.5. Chromatographic Conditions

Analyses were primarily performed on an Agilent 1100 liquid chromatographic system with a Diamond C_18_ column (4.6 mm × 250 mm, 5 *μ*m). The flow rate was 1.0 mL/min and sample injection volume was 1 *μ*L. Detection wavelength was set at 254 nm. The column temperature was maintained at 40°C. The mobile phase consisted of 0.1 mol/L acetate- (pH was adjusted to 6.5 with glacial acetic acid) acetonitrile (93 : 7) (A) and acetonitrile-water (4 : 1) (B). The gradient program was as follows: 0–2 min, 0% B; 2–5 min, 0–10% B; 15–25 min, 10–30% B; 25–33 min, 30–45% B; 33–33.1 min, 45–100% B; 33.1–36 min, 100% B; 36–36.1 min, 100–0% B; and 36.1–43 min, 0% B.

**Figure 1:**
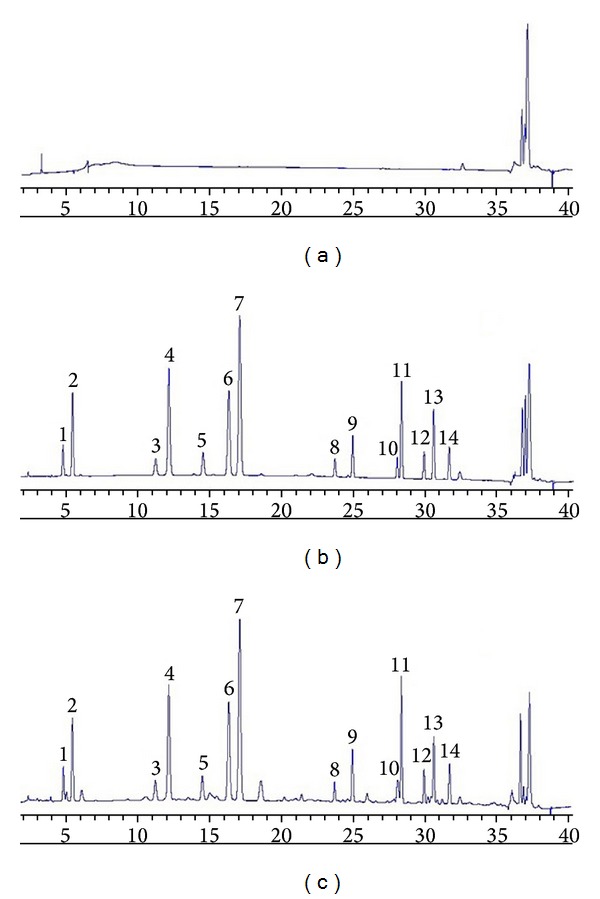
Representative HPLC chromatograms obtained from (a) the blank control solution, (b) the mixed standards solution, and (c)* QKLI* sample solution. Numbers 1–14 were identified as Asp (1), Glu (2), Ser (3), Gly (4), Arg (5), Ala (6), Pro (7), Tyr (8), Val (9), Ile (10), Leu (11), Phe (12), Orn (13), and Lys (14).

The chromatographic profiles of the blank control solution, the reference solution, and* QKLI* are shown in Figure 1. The blank control solution was obtained by preparing 0.1 mol/L hydrochloric acid according to the sample processing produce.

## 3. Principles of One for M

**Method 1 (the calibration of RCF based on external standard method)**

The absorption of analyte (peak area, *A*) was linearly proportional to sample content (concentration, *C*) in a linearity range and their relation could be shown with the formula below:

(1) A=f×C.

(1) The standard solutions of analytes were prepared and analyzed according to the HPLC conditions mentioned above; then the correction factors (CFs) were calculated by the following equation:

(2) fi=AiCi.

(2)The RCFs of each analyte relative to the selected internal standard were calculated by the formula

(3) F=Ai/CAs/Cs.

(3) When this One for M method was used, the standard solution of internal standard was reprepared and its CF calculated:

(4) fs′=As′Cs′.

The concentrations of other analytes could be calculated by their RCFs:

(5) C=Afs′×F.

**Method 2 (the calibration of RCF based on the slope of standard curve)**

(1) The standard solution of each analyte was analyzed under the conditions detailed above and established the calibration curves:

(6) A=aiC+bi,

where *A* and *C* are the peak area and concentration of compounds; *a*_*i*_ is the slope; and *b*_*i*_ is the interception.

When *a*_*i*_ is much larger than *b*_*i*_, *b*_*i*_ can be ignored and then the standard equation can be simplified into:

(7) A=aiC.

(2) The RCFs were calculated as the ratio of the equation slope for each analytes relative to the chosen internal standard

(8) Fi=aias.

(3) Reestablish the calibration curve of internal standard and obtain the slop *a*_*s*′_, and then the quantification of each sample was carried out according to the following equation:

(9) C=Aas′×F.

**Method 3 (the calibration of RCF based on the slope of standard curve and correction interception)**

(1) Established the calibration curves of each analytes, respectively:

(10) A=aiC+bi.

(2) Calculated the ratio of the slope of the linear calibration curves for each analyte relative to the internal standard as the RCFs:

(11) Fi=aias.

(3) Reestablish the calibration curve of internal standard and the contents of other components could be calculated through their RCFs and *b*_*i*_:

(12) C=A−bias′×F.

**Method 4 (the calibration of RCF based on the slope and interception of standard curve)**

(1) Develop the calibration curves of the each analyte, respectively:

(13) A=aiC+bi.

(2) Calculate the ratio of the equation slopes for each analyte relative to the internal standard as the RCFs:

(14) Fi=aias.

(3) The correction factors of interception (*B*_*i*_) were calculated as the difference of the interception for each analyte relative to the internal standard

(15) Bi=bibs.

(4) Reestablished the calibration curve of internal standard and the contents of other components were calculated as

(16) C=A−(Bi−bs′)as′×F.

There are four methods to calculate the RCFs, so four determination results of analytes can be obtained by One for M method. The choice of RCFs calculation method depends on the accuracy and the robustness, namely, the relative deviation of the analytes content between One for M method and calibration curve method.

## 4. Results and Discussion

### 4.1. Methodology Investigation on Determination of 14 AAs by Classic Calibrate Curve

#### 4.1.1. Calibration Curves, Limits of Quantification, and Detection

The stock solution of mixed standards was diluted to appropriate concentrations with 0.1 mol/mL HCl for the establishment of calibration curves. The calibration curves were obtained by plotting the ratio of peak area (*Y*) of each analyte versus the concentrations (*X*, mg/mL) of calibration standards through the linear least-squares regression analysis. All calibration curves exhibited good linearity with the correlation coefficients (*r*) higher than 0.995. The limits of quantification (LOQ) and detection (LOD) for each AA were measured based on a signal-to-noise (*S*/*N*) ratio at about 10 and 3, respectively. The data is listed in Table 1.

#### 4.1.2. Precision

Precision was evaluated by analysis of three different concentrations (low, medium, and high) with six replicates on the same day and three consecutive days. Precision was expressed as the intra- and interday relative standard deviation (RSD). The RSDs of intra- and interday were ranged from 0.75 to 2.20% and from 0.20 to 4.88%. The details are given in Table 2.

#### 4.1.3. Accuracy

The accuracy was expressed as the recovery by the standard addition method. The mixed standard solution at high, medium, and low concentration levels was added into a certain amount of the* QKLI* sample (*n* = 6); the resultant samples were processed and analyzed. The results were acceptable with the average recoveries of fourteen AAs ranged from 94.11% to 107.94% and the RSDs from 0.14 to 4.49%. All data is depicted in Table 3.

### 4.2. Relative Correction Factors (RCFs)

The four methods mentioned above were applied to calculate the RCFs with the Gly as the single standard (Table 4).

#### 4.2.1. Evaluation of RCFs

To confirm an optimal calculation method of the RCFs, six batches of* QKLI* were processed and analyzed. The Gly (standard substance) was determined directly using the calibration curve method and the content of the other 13 AAs was calculated according to their RCFs and the calibration curve, respectively. Except for Method 1, the results obtained from other three methods were satisfactory (see Supplementary Tables 1–4 available online at http://dx.doi.org/10.1155/2014/951075). Furthermore, the result of *t*-test (*P* > 0.05) indicated that there are no statistically significant differences between the calibration curve method and One for M Methods 2, 3, and 4. However, the calculation procedures of Methods 3 and 4 are more complicated for the requirement of two calculation parameters. Therefore, Method 2 was considered to be the preferred method used to calculate the RCFs.

#### 4.2.2. Robustness Validation of RCFs

The robustness test was designed to determine some potential and changeable factors on analytic procedures in different conditions.

Different instruments and columns were the most important factors to evaluate the ruggedness and robustness of RCF [33]. In our study, two instruments (Waters Alliance 2695 with PDA and Agilent 1100 with DAD) and three columns (Diamond C_18_, Phenomenex Luna C_18_, and Hypersil BDS C_18_) were used to explore. The RCFs of 13 AAs on each instrument and column are listed in Supplementary Table 5. As it showed, the RSDs of RCFs were all lower than 3.96% when analyzed on the same instrument with different columns, indicating that the different types of columns had no significant effects on RCFs. However, the RCF of Pro had a big difference in the same column on different instruments, which probably due to the methylene structure of Pro is different from others.

The RCFs were investigated at different flow rates (0.9, 1.0, 1.1 mL/min) by Agilent 1100 LC system and Diamond C_18_ (4.6 mm × 250 mm, 5 *μ*m) column. As a result, the RSDs of all AAs were within 0.24%–1.98% expected for the Asp and Ile were 2.49% and 2.78%, higher than 2% slightly. Presumably the retention time changed with the flow rates, which increasing the effect of the peak around them on their peak area. The peak area was closely related to the calculation of RCF. But the methods still met the analytical requirements with the RSDs less than 5%, indicating that the flow rate was a nonsignificant factor.

The resolution of the instrument varied with the column temperature, thereby affected the peak area of the compound, and ultimately influenced the RCF. So the different column temperatures (35°C, 40°C, and 45°C) were investigated and the RSDs ranged from 0.48% to 4.05%, revealing that the RCF was steadily under different column temperatures.

The above results showed that Method 2, the calculation method of RCF based on the slope of standard curve, had good robustness (RSD < 5%) in different instruments, columns, flow rates, and column temperatures.

Above all, Method 2 was chosen to calculate the RCFs.

### 4.3. Method Validation of One for M

To validate the One for M method, ten batches of* QKLI* were processed for HPLC analyzed. The content of the fourteen AAs was calculated (Table 5). No significant difference was found between the classic calibrate determine method and One for M method (Method 2) using the *t*-test (*P* = 95%, *P* > 0.05), indicating that the newly developed method was a satisfactory method for quality control of traditional Chinese medicines.

### 4.4. Discussion

The relative correction factor (RCF), which is the most crucial parameter in One for M method, greatly determines the accuracy of the results. Four different calculation methods of RCF were compared in the current study and the calibration method based on the slope of standard curve was selected as the best one, considering the accuracy of the results and the simplicity of the method. Therefore, the slopes of the analytes and internal standard would directly affect the accuracy of the analytic results. The slope of the standard curve can be affected by various factors, such as the purity of reference, the accuracy of measurement method, and the influence of instrument. All AA references reached enough purity (>99.5%) in this study and the accuracy of measurement method was confirmed very well. By applying different instruments, columns, and chromatographic conditions, the effect of instrument was eliminated and the robustness of RCF was also verified. The AAs must be analyzed after derivatizing due to the lack of chromophore. Phenylisothiocyanate (PITC) was selected as the derivatization reagent. The detection wavelength was set at 254 nm which is in the B absorption band of the benzene for there is no conjugation between the benzene and its side-chain of their product. The measurement results were stable and had no effect on the SCF, so the detection wavelength was not investigated in the current study. The response signals of analytes would varied with the detection wavelength when the analyres have different absorbent structure. Thus the absorption wavelength, as an important factor, need to be investigated in other researches of One for M.

In the experiment, the compound with excellent separation, high content, good stability, and suitable peak position that was in the middle of the chromatogram was preferentially selected as the internal standard. Based on the determination of AAs in several batches of* QKLI* and the calculation of RCFs, five AAs including Ser, Gly, Arg, Ala, and Pro were selected as internal standard. When the Ser and Pro were chosen as internal standard, the RDs calculated by two of four calculation methods were beyond ±5% while part of the RSD% of AAs was greater than 5% under different columns or column temperature when Arg and Ala were selected as internal standard, respectively. The four AAs mentioned above could not provide a precise result or relatively stable RCF. Eventually, Gly was used as internal standard to simultaneously determine other thirteen AAs for the satisfactory calculation results and stable RCFs.

## 5. Conclusions

Under the condition of one standard, One for M method can realize the simultaneous determination of multiple active ingredients in the herbal medicines with better reproducibility and accuracy. Using HPLC-PDA method, in the present research, fourteen AAs in ten batches of* QKLI* were quantified successfully by the One for M method. This method, with shorter analytical time, lower cost, and higher accuracy, can be employed as a rapid, practical technique to control the quality of the complex traditional herbal medicines and their preparations.

## Supplementary Material

Comparison of the amino acids content of QKLI using One for M method and traditional Calibration equation method.

## Figures and Tables

**Table 1:** Calibration curves, LOQ, and LOD of the fourteen investigated AAs.

AAs	Linear regression data			LOQ (*μ*g)	LOD (*μ*g)
	Regressive equation	Test range (mg/mL)	*r*		
Asp	*Y* = 509.433*X* − 16.066	0.050–1.000	0.9998	0.017	0.005
Glu	*Y* = 449.506*X* − 5.904	0.099–1.986	1.0000	0.040	0.012
Ser	*Y* = 642.902*X* − 4.916	0.050–1.000	0.9999	0.020	0.006
Gly	*Y* = 890.173*X* − 8.861	0.101–2.016	1.0000	0.015	0.004
Arg	*Y* = 398.247*X* − 4.785	0.050–1.005	1.0000	0.040	0.012
Ala	*Y* = 796.495*X* − 7.740	0.099–1.987	1.0000	0.030	0.009
Pro	*Y* = 659.777*X* − 6.110	0.200–4.006	0.9999	0.020	0.006
Tyr	*Y* = 419.329*X* − 3.329	0.035–0.693	0.9999	0.015	0.005
Val	*Y* = 595.691*X* − 2.190	0.050–1.002	1.0000	0.015	0.004
Ile	*Y* = 555.808*X* − 1.043	0.030–0.607	1.0000	0.010	0.003
Leu	*Y* = 560.738*X* − 1.376	0.100–1.993	1.0000	0.013	0.004
Phe	*Y* = 455.008*X* − 1.167	0.050–0.990	1.0000	0.030	0.009
Orn	*Y* = 619.760*X* − 4.322	0.050–1.010	0.9999	0.012	0.003
Lys	*Y* = 869.350*X* − 1.930	0.030–0.605	0.9999	0.010	0.003

**Table 2:** Intra- and interday precision of the fourteen investigated AAs.

AAs	Concentration (mg/mL)	Intraday (*n* = 6) RSD%	Interday (*n* = 6) RSD%	AAs	Concentration (mg/mL)	Intraday (*n* = 6) RSD%	Interday (*n* = 6) RSD%
Asp	0.101	1.63	2.11	Tyr	0.080	0.79	3.39
	0.303	2.18	2.14		0.241	1.16	4.75
	0.505	2.20	3.99		0.401	1.96	2.07

Glu	0.293	1.42	3.08	Val	0.127	1.00	3.21
	0.880	2.19	2.10		0.380	1.22	3.41
	1.467	2.14	4.36		0.634	2.11	1.15

Ser	0.069	0.75	1.34	Ile	0.063	0.84	2.21
	0.207	1.48	3.33		0.190	1.13	4.38
	0.345	2.06	2.36		0.316	2.14	2.37

Gly	0.305	0.78	2.68	Leu	0.109	0.92	2.01
	0.916	1.32	3.45		0.328	1.13	4.88
	1.527	2.02	1.60		0.547	2.04	2.50

Arg	0.141	1.23	0.20	Phe	0.166	1.02	3.58
	0.422	1.74	2.86		0.498	0.98	2.01
	0.703	1.90	4.09		0.831	1.98	4.41

Ala	0.303	0.84	3.74	Orn	0.300	1.08	3.96
	0.909	1.25	3.34		0.901	0.96	3.23
	1.514	2.06	1.29		1.501	1.97	4.02

Pro	0.655	0.92	4.23	Lys	0.076	1.21	3.91
	1.964	1.36	3.42		0.228	0.92	2.57
	3.274	2.06	1.65		0.380	2.02	4.47

**Table 3:** The average recoveries of the fourteen investigated AAs (*n* = 6).

Analytes	Original (mg)	Spiked (mg)	Found (mg)	Recovery^a^ (%)	RSD (%)
Asp	0.149	0.100	0.256	106.72	2.41
		0.200	0.365	107.94	2.39
		0.300	0.460	103.56	3.25

Glu	0.531	0.291	0.830	102.84	4.49
		0.582	1.152	106.73	1.96
		0.873	1.471	107.60	2.44

Ser	0.148	0.070	0.217	97.76	1.25
		0.141	0.281	94.50	0.14
		0.211	0.371	105.44	4.23

Gly	0.632	0.305	0.945	103.02	0.26
		0.609	1.266	104.16	2.57
		0.914	1.603	106.32	1.10

Arg	0.264	0.140	0.412	105.39	2.51
		0.281	0.544	99.58	1.53
		0.421	0.714	106.89	1.10

Ala	0.622	0.303	0.942	105.61	2.79
		0.606	1.270	107.05	3.70
		0.909	1.581	105.57	1.90

Pro	1.445	0.656	2.102	100.17	2.63
		1.313	2.829	105.48	1.04
		1.969	3.721	107.69	2.50

Tyr	0.136	0.079	0.219	105.27	2.95
		0.159	0.291	98.17	2.71
		0.238	0.384	104.35	3.80

Val	0.273	0.128	0.407	104.87	3.85
		0.256	0.543	105.62	1.50
		0.384	0.683	106.90	3.01

Ile	0.115	0.063	0.181	103.84	2.92
		0.127	0.236	95.68	0.94
		0.190	0.320	107.80	3.71

Leu	0.607	0.297	0.908	101.73	2.15
		0.593	1.221	103.56	1.25
		0.890	1.517	102.30	3.68

Phe	0.233	0.110	0.338	94.87	4.12
		0.220	0.441	94.11	2.16
		0.331	0.586	106.77	2.06

Orn	0.200	0.166	0.371	103.11	2.24
		0.332	0.556	107.18	1.07
		0.497	0.726	105.64	3.94

Lys	0.133	0.076	0.214	106.01	3.91
		0.153	0.291	103.14	2.19
		0.229	0.374	105.37	2.05

^a^Recovery (%) = 100 × (found amount − original amount)/spiked amount; the data presented as average of three determinations.

**Table 4:** Relative correction factors (RCFs) of the 13 AAs.

AAs	Relative response factors					
	Method I	Method II	Method III		Method IV	
	*F*	*F*	*F*	bi	*F*	Bi
Asp	0.531	0.551	0.551	−0.466	0.551	−9.425
Glu	0.492	0.501	0.501	1.498	0.501	−7.462
Ser	0.662	0.696	0.696	−1.768	0.696	−10.728
Arg	0.441	0.450	0.450	0.744	0.450	−8.215
Ala	0.896	0.891	0.891	8.858	0.891	−0.101
Pro	0.749	0.730	0.730	20.829	0.730	11.870
Tyr	0.471	0.470	0.470	1.501	0.470	−7.458
Val	0.677	0.666	0.666	4.178	0.666	−4.781
Ile	0.637	0.629	0.629	1.882	0.629	−7.077
Leu	0.646	0.633	0.633	8.324	0.633	−0.636
Phe	0.533	0.514	0.514	4.430	0.514	−4.529
Orn	0.913	0.932	0.932	1.226	0.932	−7.733
Lys	0.982	0.987	0.987	2.056	0.987	−6.904

**Table 5:** Contents of 14 AAs in ten batches of *QKLI* by two methods.

AAs	013105A		RD^a^ (%)	110101A		RD^a^ (%)	115202A		RD^a^ (%)	210204A		RD^a^ (%)	111103A		RD^a^ (%)
	a	b		a	b		a	b		a	b		a	b	
Gly	—	1.039	—	—	1.028	—	—	0.997	—	—	0.983	—	—	1.046	—
Asp	0.273	0.269	1.29	0.324	0.321	0.81	0.315	0.312	0.88	0.338	0.336	0.70	0.339	0.337	0.69
Glu	1.055	1.074	−1.74	1.008	1.026	−1.72	0.922	0.937	−1.67	0.954	0.970	−1.69	0.996	1.013	−1.71
Ser	0.284	0.284	−0.04	0.296	0.296	−0.04	0.288	0.288	−0.04	0.254	0.254	−0.03	0.273	0.273	−0.04
Arg	0.424	0.429	−1.19	0.434	0.440	−1.20	0.711	0.720	−1.31	0.661	0.670	−1.30	0.611	0.619	−1.28
Ala	1.200	1.211	−0.86	1.133	1.142	−0.81	1.074	1.082	−0.76	1.078	1.087	−0.77	1.180	1.190	−0.85
Pro	2.284	2.254	1.34	2.471	2.440	1.26	2.179	2.149	1.38	2.357	2.326	1.31	2.832	2.800	1.15
Tyr	0.234	0.233	0.57	0.270	0.269	0.39	0.251	0.250	0.48	0.196	0.194	0.83	0.237	0.236	0.56
Val	0.537	0.542	−0.91	0.511	0.515	−0.83	0.483	0.486	−0.73	0.506	0.510	−0.81	0.555	0.560	−0.95
Ile	0.264	0.263	0.32	0.249	0.248	0.49	0.232	0.231	0.72	0.246	0.245	0.53	0.273	0.272	0.23
Leu	1.209	1.212	−0.24	1.124	1.126	−0.15	1.075	1.075	−0.08	1.070	1.071	−0.08	1.185	1.188	−0.22
Trp	0.408	0.403	1.20	0.377	0.372	1.35	0.368	0.362	1.40	0.378	0.373	1.34	0.404	0.399	1.22
Orn	0.353	0.355	−0.58	0.322	0.324	−0.63	0.323	0.325	−0.63	0.314	0.316	−0.64	0.328	0.330	−0.62
Lys	0.245	0.254	−3.50	0.218	0.226	−3.41	0.234	0.243	−3.46	0.228	0.236	−3.44	0.244	0.253	−3.50

AAs	115203A		RD^a^ (%)	210401A		RD^a^ (%)	012602A		RD^a^ (%)	210301A		RD^a^ (%)	115702A		RD^a^ (%)
	a	b		a	b		a	b		a	b		a	b	

Gly	—	1.077	—	—	1.115	—	—	1.145	—	—	1.125	—	—	1.138	—
Asp	0.359	0.357	0.55	0.356	0.354	0.57	0.375	0.373	0.46	0.372	0.370	0.48	0.397	0.395	0.34
Glu	1.016	1.034	−1.72	0.978	0.995	−1.70	0.966	0.983	−1.70	0.997	1.015	−1.71	1.094	1.114	−1.76
Ser	0.332	0.332	−0.06	0.301	0.301	−0.05	0.275	0.275	−0.04	0.279	0.279	−0.04	0.324	0.324	−0.05
Arg	0.821	0.832	−1.34	0.659	0.668	−1.30	0.596	0.604	−1.28	0.483	0.489	−1.23	0.576	0.583	−1.27
Ala	1.167	1.177	−0.84	1.199	1.209	−0.86	1.233	1.244	−0.88	1.184	1.194	−0.85	1.254	1.265	−0.90
Pro	2.367	2.337	1.30	2.489	2.458	1.26	2.919	2.886	1.13	2.251	2.221	1.35	2.225	2.195	1.36
Tyr	0.257	0.256	0.45	0.217	0.216	0.68	0.289	0.288	0.32	0.213	0.211	0.71	0.247	0.246	0.50
Val	0.525	0.529	−0.87	0.538	0.543	−0.91	0.530	0.535	−0.88	0.533	0.537	−0.89	0.566	0.571	−0.98
Ile	0.251	0.250	0.47	0.259	0.258	0.37	0.258	0.257	0.39	0.263	0.262	0.33	0.279	0.278	0.17
Leu	1.161	1.163	−0.19	1.166	1.168	−0.19	1.205	1.208	−0.24	1.167	1.170	−0.20	1.243	1.246	−0.27
Trp	0.390	0.385	1.29	0.393	0.388	1.27	0.408	0.403	1.20	0.408	0.403	1.20	0.431	0.426	1.11
Orn	0.362	0.365	−0.56	0.349	0.351	−0.58	0.366	0.368	−0.56	0.380	0.382	−0.54	0.382	0.384	−0.53
Lys	0.257	0.267	−3.53	0.250	0.259	−3.51	0.257	0.267	−3.53	0.265	0.275	−3.56	0.268	0.278	−3.56

^a^Relative deviation = (a − b)/b.

a: the content were calculated by One for M method; b: the content were determined by the traditional calibration equation method.
