# Supplementary material for: One Single Amino Acid for Estimation the Content of Total Free Amino Acids in Qingkailing Injection Using High-Performance Liquid Chromatography-Diode Array Detection
Source: J Anal Methods Chem. 2014 Mar 13;2014:951075. doi: 10.1155/2014/951075 (PMC3976834; doi:10.1155/2014/951075)
Supplement: Supplementary file 1 — Comparison of the amino acids content of QKLI using One for M method and traditional Calibration equation method. [file 951075.f1.pdf]

*Supplementary materials*

# One single amino acid for estimation the content of total free amino acids in *Qingkailing injection* using high-performance liquid chromatography-diode array detection

Li Zhang[a], Haiyu Liu[a], Long Peng[a], Mingxing Guo[a], Zhixin Zhang[a], Lingling Qin[a], Xiaoyan Gao[a,]*

[a] Science Experiment Center for Traditional Chinese Medicine, Beijing University of Chinese Medicine, No. 11, North Third Ring Road, Chaoyang District, Beijing 100029, P. R. China

* Corresponding author: Xiaoyan Gao, Science Experiment Center for Traditional Chinese Medicine, Beijing University of Chinese Medicine, No. 11, North Third Ring Road, Chaoyang District, Beijing 100029, P. R. China, Tel: +86 010 64286401; Fax: +86 010 64286052; E-mail: gaoxiaoyan0913@sina.com

# Supplementary materials

**Table S-1.** Comparison of AAs content determined by two methods( Method I**)**

**Table S-2.** Comparison of AAs content determined by two methods( Method II**)**

**Table S-3.** Comparison of AAs content determined by two methods( Method III**)**

**Table S-4.** Comparison of AAs content determined by two methods( Method IV**)**

**Table S-5.** RCFs in different instruments and columns

Table S1: Contents of 14 AAs in different batches of *QKLI*

| AAs | 113901A | | RD [a] | 110701A | | RD [a] | 111203A | | RD [a] | 110505A | | RD [a] | 010705A | | RD [a] | 012907A | | RD [a] |
|---|---|---|---|---|---|---|---|---|---|---|---|---|---|---|---|---|---|---|
| | a | b | (%) | a | b | (%) | a | b | (%) | a | b | (%) | a | b | (%) | a | b | (%) |
| Gly | - | 1.100 | - | - | 1.041 | - | - | 1.126 | - | - | 1.013 | - | - | 1.274 | - | - | 1.073 | - |
| Asp | 0.389 | 0.384 | 1.31 | 0.325 | 0.319 | 1.74 | 0.336 | 0.331 | 1.65 | 0.314 | 0.308 | 1.83 | 0.450 | 0.446 | 1.02 | 0.329 | 0.323 | 1.71 |
| Glu | 1.031 | 1.060 | -2.70 | 1.008 | 1.036 | -2.69 | 1.052 | 1.081 | -2.71 | 0.987 | 1.014 | -2.68 | 1.311 | 1.349 | -2.81 | 1.066 | 1.096 | -2.72 |
| Ser | 0.288 | 0.282 | 2.17 | 0.282 | 0.276 | 2.17 | 0.307 | 0.301 | 2.16 | 0.285 | 0.279 | 2.17 | 0.324 | 0.317 | 2.15 | 0.248 | 0.242 | 2.18 |
| Arg | 0.708 | 0.722 | -1.97 | 0.511 | 0.520 | -1.90 | 0.487 | 0.496 | -1.89 | 0.462 | 0.471 | -1.88 | 0.654 | 0.667 | -1.96 | 0.595 | 0.607 | -1.94 |
| Ala | 1.122 | 1.169 | -4.06 | 1.127 | 1.175 | -4.06 | 1.174 | 1.224 | -4.09 | 1.120 | 1.167 | -4.05 | 1.262 | 1.316 | -4.15 | 1.111 | 1.158 | -4.05 |
| Pro | 2.381 | 2.484 | -4.14 | 2.088 | 2.176 | -4.03 | 2.687 | 2.806 | -4.23 | 2.095 | 2.183 | -4.03 | 2.788 | 2.912 | -4.26 | 2.398 | 2.501 | -4.15 |
| Tyr | 0.23 | 0.23 | -2.58 | 0.25 | 0.25 | -2.68 | 0.24 | 0.25 | -2.67 | 0.23 | 0.24 | -2.62 | 0.28 | 0.29 | -2.81 | 0.25 | 0.25 | -2.67 |

| | | | | | | | | | | | | | | | | | | |
|---|---|---|---|---|---|---|---|---|---|---|---|---|---|---|---|---|---|---|
| | 2 | 8 | | 0 | 7 | | 8 | 5 | | 9 | 6 | | 3 | 1 | | 0 | 7 | |
| Val | 0.516 | 0.544 | -5.19 | 0.503 | 0.530 | -5.16 | 0.531 | 0.560 | -5.24 | 0.492 | 0.519 | -5.12 | 0.529 | 0.558 | -5.23 | 0.493 | 0.520 | -5.13 |
| Ile | 0.254 | 0.264 | -3.78 | 0.246 | 0.256 | -3.69 | 0.262 | 0.272 | -3.86 | 0.242 | 0.251 | -3.64 | 0.264 | 0.275 | -3.88 | 0.251 | 0.261 | -3.74 |
| Leu | 1.127 | 1.187 | -4.99 | 1.119 | 1.177 | -4.98 | 1.201 | 1.265 | -5.06 | 1.081 | 1.137 | -4.94 | 1.241 | 1.308 | -5.10 | 1.120 | 1.179 | -4.98 |
| Phe | 0.370 | 0.389 | -5.08 | 0.372 | 0.392 | -5.09 | 0.406 | 0.428 | -5.23 | 0.355 | 0.374 | -5.01 | 0.431 | 0.455 | -5.32 | 0.375 | 0.396 | -5.11 |
| Orn | 0.326 | 0.330 | -1.32 | 0.328 | 0.332 | -1.31 | 0.325 | 0.329 | -1.32 | 0.303 | 0.307 | -1.36 | 0.521 | 0.527 | -1.10 | 0.359 | 0.363 | -1.26 |
| Lys | 0.240 | 0.254 | -5.72 | 0.235 | 0.250 | -5.71 | 0.228 | 0.242 | -5.68 | 0.213 | 0.226 | -5.63 | 0.319 | 0.339 | -5.90 | 0.247 | 0.262 | -5.74 |

[a] relative deviation=(a-b)/b

a: the content were calculated by One for M method ; b: the content were determined by the traditional Calibration equation method

Table S2: Contents of 14 AAs in different batches of *QKLI*

| AAs | 113901A | | RD [a] | 110701A | | RD [a] | 111203A | | RD [a] | 110505A | | RD [a] | 010705A | | RD [a] | 012907A | | RD [a] |
|---|---|---|---|---|---|---|---|---|---|---|---|---|---|---|---|---|---|---|
| | a | b | (%) | a | b | (%) | a | b | (%) | a | b | (%) | a | b | (%) | a | b | (%) |
| Gly | - | 1.100 | - | - | 1.041 | - | - | 1.126 | - | - | 1.013 | - | - | 1.274 | - | - | 1.073 | - |
| Asp | 0.385 | 0.384 | 0.40 | 0.322 | 0.319 | 0.82 | 0.333 | 0.331 | 0.74 | 0.311 | 0.308 | 0.91 | 0.446 | 0.446 | 0.11 | 0.326 | 0.323 | 0.79 |
| Glu | 1.041 | 1.060 | -1.74 | 1.018 | 1.036 | -1.73 | 1.062 | 1.081 | -1.75 | 0.997 | 1.014 | -1.71 | 1.324 | 1.349 | -1.84 | 1.076 | 1.096 | -1.75 |
| Ser | 0.282 | 0.282 | -0.04 | 0.276 | 0.276 | -0.04 | 0.301 | 0.301 | -0.05 | 0.279 | 0.279 | -0.04 | 0.317 | 0.317 | -0.05 | 0.242 | 0.242 | -0.02 |
| Arg | 0.713 | 0.722 | -1.31 | 0.514 | 0.520 | -1.25 | 0.490 | 0.496 | -1.23 | 0.466 | 0.471 | -1.22 | 0.658 | 0.667 | -1.30 | 0.599 | 0.607 | -1.28 |
| Ala | 1.159 | 1.169 | -0.83 | 1.165 | 1.175 | -0.84 | 1.213 | 1.224 | -0.87 | 1.157 | 1.167 | -0.83 | 1.304 | 1.316 | -0.93 | 1.148 | 1.158 | -0.82 |
| Pro | 2.515 | 2.484 | 1.25 | 2.206 | 2.176 | 1.37 | 2.838 | 2.806 | 1.15 | 2.213 | 2.183 | 1.37 | 2.945 | 2.912 | 1.13 | 2.532 | 2.501 | 1.24 |
| Tyr | 0.24 | 0.23 | 0.54 | 0.25 | 0.25 | 0.45 | 0.25 | 0.25 | 0.46 | 0.24 | 0.24 | 0.50 | 0.29 | 0.29 | 0.31 | 0.25 | 0.25 | 0.45 |

| | | | | | | | | | | | | | | | | | | |
|---|---|---|---|---|---|---|---|---|---|---|---|---|---|---|---|---|---|---|
| | 0 | 8 | | 9 | 7 | | 6 | 5 | | 7 | 6 | | 2 | 1 | | 8 | 7 | |
| Val | 0.539 | 0.544 | -0.91 | 0.525 | 0.530 | -0.87 | 0.555 | 0.560 | -0.95 | 0.514 | 0.519 | -0.84 | 0.553 | 0.558 | -0.95 | 0.515 | 0.520 | -0.84 |
| Ile | 0.265 | 0.264 | 0.31 | 0.257 | 0.256 | 0.40 | 0.273 | 0.272 | 0.22 | 0.253 | 0.251 | 0.45 | 0.276 | 0.275 | 0.20 | 0.261 | 0.261 | 0.35 |
| Leu | 1.184 | 1.187 | -0.21 | 1.175 | 1.177 | -0.20 | 1.261 | 1.265 | -0.29 | 1.135 | 1.137 | -0.16 | 1.303 | 1.308 | -0.33 | 1.176 | 1.179 | -0.21 |
| Phe | 0.394 | 0.389 | 1.26 | 0.397 | 0.392 | 1.25 | 0.433 | 0.428 | 1.10 | 0.379 | 0.374 | 1.34 | 0.460 | 0.455 | 1.01 | 0.400 | 0.396 | 1.24 |
| Orn | 0.328 | 0.330 | -0.62 | 0.330 | 0.332 | -0.61 | 0.327 | 0.329 | -0.62 | 0.305 | 0.307 | -0.66 | 0.525 | 0.527 | -0.40 | 0.361 | 0.363 | -0.56 |
| Lys | 0.245 | 0.254 | -3.50 | 0.241 | 0.250 | -3.49 | 0.233 | 0.242 | -3.46 | 0.218 | 0.226 | -3.41 | 0.326 | 0.339 | -3.69 | 0.253 | 0.262 | -3.52 |

[a] relative deviation=(a-b)/b

a: the content were calculated by One for M method ; b: the content were determined by the traditional Calibration equation method

Table S3: Contents of 14 AAs in different batches of *QKLI*

| AAs | 113901A | | RD [a] | 110701A | | RD [a] | 111203A | | RD [a] | 110505A | | RD [a] | 010705A | | RD [a] | 012907A | | RD [a] |
|---|---|---|---|---|---|---|---|---|---|---|---|---|---|---|---|---|---|---|
| | a | b | (%) | a | b | (%) | a | b | (%) | a | b | (%) | a | b | (%) | a | b | (%) |
| Gly | - | 1.100 | - | - | 1.041 | - | - | 1.126 | - | - | 1.013 | - | - | 1.274 | - | - | 1.073 | - |
| Asp | 0.386 | 0.384 | 0.69 | 0.323 | 0.319 | 1.17 | 0.334 | 0.331 | 1.07 | 0.312 | 0.308 | 1.28 | 0.447 | 0.446 | 0.36 | 0.327 | 0.323 | 1.13 |
| Glu | 1.037 | 1.060 | -2.11 | 1.014 | 1.036 | -2.11 | 1.058 | 1.081 | -2.11 | 0.993 | 1.014 | -2.10 | 1.320 | 1.349 | -2.14 | 1.073 | 1.096 | -2.11 |
| Ser | 0.286 | 0.282 | 1.15 | 0.279 | 0.276 | 1.17 | 0.304 | 0.301 | 1.07 | 0.282 | 0.279 | 1.16 | 0.320 | 0.317 | 1.00 | 0.246 | 0.242 | 1.36 |
| Arg | 0.711 | 0.722 | -1.62 | 0.512 | 0.520 | -1.66 | 0.488 | 0.496 | -1.67 | 0.463 | 0.471 | -1.68 | 0.656 | 0.667 | -1.63 | 0.597 | 0.607 | -1.64 |
| Ala | 1.146 | 1.169 | -1.95 | 1.152 | 1.175 | -1.95 | 1.200 | 1.224 | -1.94 | 1.144 | 1.167 | -1.95 | 1.291 | 1.316 | -1.92 | 1.135 | 1.158 | -1.96 |
| Pro | 2.477 | 2.484 | -0.26 | 2.168 | 2.176 | -0.36 | 2.801 | 2.806 | -0.19 | 2.176 | 2.183 | -0.36 | 2.907 | 2.912 | -0.17 | 2.495 | 2.501 | -0.26 |
| Tyr | 0.23 | 0.23 | -1.23 | 0.25 | 0.25 | -1.19 | 0.25 | 0.25 | -1.19 | 0.24 | 0.24 | -1.21 | 0.28 | 0.29 | -1.14 | 0.25 | 0.25 | -1.19 |

| | | | | | | | | | | | | | | | | | | |
|---|---|---|---|---|---|---|---|---|---|---|---|---|---|---|---|---|---|---|
| | 6 | 8 | | 4 | 7 | | 2 | 5 | | 3 | 6 | | 8 | 1 | | 4 | 7 | |
| Val | 0.531 | 0.544 | -2.43 | 0.517 | 0.530 | -2.43 | 0.547 | 0.560 | -2.43 | 0.506 | 0.519 | -2.43 | 0.545 | 0.558 | -2.43 | 0.507 | 0.520 | -2.43 |
| Ile | 0.261 | 0.264 | -1.18 | 0.253 | 0.256 | -1.14 | 0.269 | 0.272 | -1.22 | 0.249 | 0.251 | -1.12 | 0.272 | 0.275 | -1.23 | 0.258 | 0.261 | -1.17 |
| Leu | 1.167 | 1.187 | -1.67 | 1.158 | 1.177 | -1.68 | 1.244 | 1.265 | -1.66 | 1.118 | 1.137 | -1.68 | 1.286 | 1.308 | -1.66 | 1.159 | 1.179 | -1.68 |
| Phe | 0.383 | 0.389 | -1.65 | 0.386 | 0.392 | -1.64 | 0.421 | 0.428 | -1.55 | 0.368 | 0.374 | -1.70 | 0.448 | 0.455 | -1.49 | 0.389 | 0.396 | -1.64 |
| Orn | 0.327 | 0.330 | -1.14 | 0.329 | 0.332 | -1.14 | 0.326 | 0.329 | -1.15 | 0.304 | 0.307 | -1.23 | 0.523 | 0.527 | -0.73 | 0.360 | 0.363 | -1.04 |
| Lys | 0.243 | 0.254 | -4.58 | 0.238 | 0.250 | -4.59 | 0.231 | 0.242 | -4.60 | 0.216 | 0.226 | -4.62 | 0.323 | 0.339 | -4.50 | 0.250 | 0.262 | -4.57 |

[a] relative deviation=(a-b)/b

a: the content were calculated by One for M method ; b: the content were determined by the traditional Calibration equation method

Table S4: Contents of 14 AAs in different batches of *QKLI*

| AAs | 113901A | | RD [a] | 110701A | | RD [a] | 111203A | | RD [a] | 110505A | | RD [a] | 010705A | | RD [a] | 012907A | | RD [a] |
|---|---|---|---|---|---|---|---|---|---|---|---|---|---|---|---|---|---|---|
| | a | b | (%) | a | b | (%) | a | b | (%) | a | b | (%) | a | b | (%) | a | b | (%) |
| Gly | - | 1.100 | - | - | 1.041 | - | - | 1.126 | - | - | 1.013 | - | - | 1.274 | - | - | 1.073 | - |
| Asp | 0.392 | 0.384 | 2.19 | 0.329 | 0.319 | 2.98 | 0.340 | 0.331 | 2.82 | 0.318 | 0.308 | 3.15 | 0.453 | 0.446 | 1.65 | 0.333 | 0.323 | 2.92 |
| Glu | 1.044 | 1.060 | -1.51 | 1.021 | 1.036 | -1.49 | 1.065 | 1.081 | -1.52 | 0.999 | 1.014 | -1.48 | 1.327 | 1.349 | -1.67 | 1.079 | 1.096 | -1.53 |
| Ser | 0.290 | 0.282 | 2.77 | 0.284 | 0.276 | 2.83 | 0.308 | 0.301 | 2.59 | 0.287 | 0.279 | 2.80 | 0.325 | 0.317 | 2.45 | 0.250 | 0.242 | 3.25 |
| Arg | 0.718 | 0.722 | -0.64 | 0.519 | 0.520 | -0.30 | 0.495 | 0.496 | -0.25 | 0.470 | 0.471 | -0.18 | 0.663 | 0.667 | -0.56 | 0.604 | 0.607 | -0.47 |
| Ala | 1.150 | 1.169 | -1.65 | 1.156 | 1.175 | -1.65 | 1.204 | 1.224 | -1.65 | 1.148 | 1.167 | -1.65 | 1.295 | 1.316 | -1.65 | 1.139 | 1.158 | -1.65 |
| Pro | 2.48 | 2.48 | -0.09 | 2.17 | 2.17 | -0.16 | 2.80 | 2.80 | -0.03 | 2.18 | 2.18 | -0.16 | 2.91 | 2.91 | -0.02 | 2.49 | 2.50 | -0.09 |

| | | | | | | | | | | | | | | | | | | |
|---|---|---|---|---|---|---|---|---|---|---|---|---|---|---|---|---|---|---|
| | 1 | 4 | | 3 | 6 | | 5 | 6 | | 0 | 3 | | 2 | 2 | | 9 | 1 | |
| Tyr | 0.242 | 0.238 | 1.62 | 0.261 | 0.257 | 1.45 | 0.259 | 0.255 | 1.46 | 0.249 | 0.246 | 1.55 | 0.295 | 0.291 | 1.19 | 0.261 | 0.257 | 1.45 |
| Val | 0.535 | 0.544 | -1.55 | 0.522 | 0.530 | -1.53 | 0.551 | 0.560 | -1.57 | 0.511 | 0.519 | -1.51 | 0.550 | 0.558 | -1.57 | 0.512 | 0.520 | -1.51 |
| Ile | 0.266 | 0.264 | 0.74 | 0.258 | 0.256 | 0.84 | 0.274 | 0.272 | 0.64 | 0.254 | 0.251 | 0.90 | 0.277 | 0.275 | 0.61 | 0.263 | 0.261 | 0.78 |
| Leu | 1.172 | 1.187 | -1.25 | 1.163 | 1.177 | -1.25 | 1.249 | 1.265 | -1.26 | 1.123 | 1.137 | -1.24 | 1.291 | 1.308 | -1.27 | 1.164 | 1.179 | -1.25 |
| Phe | 0.389 | 0.389 | -0.06 | 0.392 | 0.392 | -0.06 | 0.427 | 0.428 | -0.10 | 0.374 | 0.374 | -0.04 | 0.454 | 0.455 | -0.13 | 0.395 | 0.396 | -0.07 |
| Orn | 0.330 | 0.330 | -0.11 | 0.332 | 0.332 | -0.11 | 0.329 | 0.329 | -0.11 | 0.307 | 0.307 | -0.11 | 0.526 | 0.527 | -0.08 | 0.363 | 0.363 | -0.10 |
| Lys | 0.246 | 0.254 | -3.31 | 0.241 | 0.250 | -3.29 | 0.234 | 0.242 | -3.26 | 0.219 | 0.226 | -3.19 | 0.327 | 0.339 | -3.54 | 0.254 | 0.262 | -3.34 |

[a] relative deviation=(a-b)/b

a: the content were calculated by One for M method ; b: the content were determined by the traditional Calibration equation method

Table S5: RCFs in different instruments and columns

| AAs | Agilent | | | RSD % | Wasters | | | RSD % |
|---|---|---|---|---|---|---|---|---|
| | luna column | Hypersil column | Diamonsi column | | luna column | Hypersil column | Diamonsi column | |
| Asp | 0.564 | 0.560 | 0.542 | 2.14 | 0.548 | 0.545 | 0.572 | 2.67 |
| Glu | 0.502 | 0.497 | 0.490 | 1.23 | 0.483 | 0.488 | 0.507 | 2.53 |
| Ser | 0.691 | 0.688 | 0.695 | 0.51 | 0.693 | 0.690 | 0.693 | 0.24 |
| Arg | 0.452 | 0.441 | 0.444 | 1.23 | 0.456 | 0.451 | 0.471 | 2.27 |
| Ala | 0.890 | 0.881 | 0.876 | 0.80 | 0.885 | 0.877 | 0.885 | 0.48 |
| Pro | 0.734 | 0.724 | 0.733 | 0.74 | 0.685 | 0.673 | 0.683 | 0.91 |
| Tyr | 0.468 | 0.464 | 0.466 | 0.37 | 0.472 | 0.468 | 0.470 | 0.41 |
| Val | 0.674 | 0.663 | 0.650 | 1.80 | 0.680 | 0.673 | 0.678 | 0.52 |
| Ile | 0.631 | 0.647 | 0.613 | 2.68 | 0.655 | 0.655 | 0.611 | 3.96 |
| Leu | 0.633 | 0.637 | 0.623 | 1.11 | 0.641 | 0.639 | 0.642 | 0.23 |
| Phe | 0.512 | 0.512 | 0.511 | 0.14 | 0.520 | 0.518 | 0.517 | 0.25 |
| Orn | 0.935 | 0.929 | 0.932 | 0.33 | 0.940 | 0.931 | 0.941 | 0.57 |
| Lys | 0.972 | 0.977 | 0.945 | 1.82 | 0.975 | 0.977 | 0.988 | 0.75 |
